# Supplementary material for: Body Fat Reduction Effect of Bifidobacterium breve B-3: A Randomized, Double-Blind, Placebo Comparative Clinical Trial
Source: Nutrients. 2022 Dec 21;15(1):28. doi: 10.3390/nu15010028 (PMC9824586; doi:10.3390/nu15010028)
Supplement: Supplementary file 1 [file nutrients-15-00028-s001.zip › nutrients-2046908-supplementary.pdf]

Table S1. Raw materials and mixing ratio of the study intervention

	Raw material	Mixing ratio (%)	Content (mg)
BB-3	*Bifidobacterium breve* B-3 *	11.11	50.00
	Maltodextrin	85.89	386.50
	Magnesium stearate	1.00	4.50
	Silicon dioxide	2.00	9.00
	Total	100.00	450.00
Placebo	Maltodextrin	97.00	436.50
	Magnesium stearate	1.00	4.50
	Silicon dioxide	2.00	9.00
	Total	100.00	450.00

**Bifidobacterium breve* B-3 contains 1×10^{11} CFU of *Bifidobacterium breve* B-3 per 1 g of corn starch including *Bifidobacterium breve* B-3 as the same raw material as the B-3-EX product sold commercially by Morinaga Dairy in Japan. Every 50 mg of *Bifidobacterium breve* contains 5×10^{9} CFU of *Bifidobacterium breve* B-3.

Table S2. Changes in body weight and body mass index (PPS)

		BB-3 group (N=42)	Placebo group (N=41)
Body weight (kg), mean±SD	V2	72.71±8.22	70.82±8.17
	V5	71.48±7.80	70.67±8.25
	V5-V2	-1.23±1.83	-0.15±1.49
	p-value	<0.0001[2]	0.5332[1]
	Difference V5-V2 (Tx-Px)	-1.08±1.67	
	p-value	0.0060[3]	
Body mass index (kg/m^2), mean±SD	V2	26.93±1.29	26.85±1.38
	V5	26.49±1.34	26.79±1.51
	V5-V2	-0.43±0.64	-0.06±0.57
	p-value	0.0001[1]	0.5344[1]
	Difference V5-V2 (Tx-Px)	-0.38±0.61	
	LS mean difference	-0.37	
	p-value	0.0074[4]	

[1] p-value for the paired t-test (T)
[2] p-value for the Wilcoxon signed rank test (W)
[3] p-value for the Wilcoxon rank sum test
[4] p-value for ANCOVA adjusted for baseline values and sex

Table S3. Changes in waist circumference, hip circumference, and waist-hip-ratio (PPS)

		BB-3 group (N=42)	Placebo group (N=41)
Waist circumference (cm), mean±SD	V2	88.39±4.58	87.62±5.55
	V5	86.39±4.15	87.86±5.50
	V5-V2	-2.01±3.12	0.23±3.95
	p-value	0.0002[1]	0.7423[2]
	Difference V5-V2 (Tx-Px)	-2.24±3.55	
	p-value	0.0042[3]	
Hip circumference (cm), mean±SD	V2	99.04±3.66	98.60±3.89
	V5	97.96±3.35	98.58±3.56
	V5-V2	-1.08±2.50	-0.02±2.20
	p-value	0.0011[2]	0.9493[1]
	Difference V5-V2 (Tx-Px)	-1.06±2.35	
	p-value	0.0161[3]	
Waist:hip ratio, mean±SD	V2	0.89±0.04	0.89±0.05
	V5	0.88±0.04	0.89±0.05
	V5-V2	-0.01±0.03	0.00±0.04
	p-value	0.0468[2]	0.6571[1]
	Difference V5-V2 (Tx-Px)	-0.01±0.03	
	p-value	0.1861[3]	

[1] p-value for the paired t-test (T)

[2] p-value for the Wilcoxon signed rank test (W)

[3] p-value for the Wilcoxon rank sum test

Table S4. DEXA results by anatomical region at 12 weeks from baseline (PPS)

			BB-3 group (N=42)	Placebo group (N=41)
Body fat mass (g), mean±SD	Arm	V2	2717.45±451.18	2851.51±570.51
		V5	2684.07±525.71	2854.12±532.35
		V5-V2	-33.38±303.75	2.61±212.80
		p-value	0.3292[2]	0.9378[1]
		Difference V5-V2 (Tx-Px)	-35.99±262.80	
		p-value	0.4046[4]	
	Legs	V2	7171.36±1802.64	7672.22±1758.44
		V5	7004.48±1765.71	7588.22±1831.60
		V5-V2	-166.88±403.76	-84.00±339.32
		p-value	0.0106[1]	0.1208[1]
		Difference V5-V2 (Tx-Px)	-82.88±373.33	
		LS mean difference	-90.29	
		p-value	0.2834[3]	
	Trunk	V2	14606.17±2651.25	14693.63±2329.44
		V5	14224.12±2643.83	14710.20±2390.93
		V5-V2	-382.05±738.20	16.56±700.23
		p-value	0.0017[1]	0.8804[1]
		Difference V5-V2 (Tx-Px)	-398.61±719.70	
		LS mean difference	-399.78	
		p-value	0.0144[3]	
	Android	V2	2353.33±501.31	2381.15±488.41
		V5	2268.76±467.66	2392.17±486.80
		V5-V2	-84.57±184.00	11.02±148.78
		p-value	0.0048[1]	0.6378[1]
		Difference V5-V2 (Tx-Px)	-95.60±167.54	

		LS mean difference	-93.27	
		p-value	0.0104[3]	
	Gynoid	V2	3884.48±977.53	4112.54±979.29
		V5	3818.45±1008.41	4150.12±929.63
		V5-V2	-66.02±149.31	37.59±526.11
		p-value	0.0065[1]	0.5925[2]
		Difference V5-V2 (Tx-Px)	-103.61±384.67	
		p-value	0.4202[4]	
Body fat percentage (%), mean±SD	Arm	V2	36.37±8.45	39.40±8.51
		V5	36.16±8.88	39.29±8.22
		V5-V2	-0.21±2.02	-0.11±1.71
		p-value	0.5095[1]	0.9400[2]
		Difference V5-V2 (Tx-Px)	-0.10±1.87	
		p-value	0.5060[4]	
	Legs	V2	33.05±7.71	35.80±7.53
		V5	32.85±7.70	35.53±7.65
		V52-V2	-0.20±1.20	-0.27±1.15
		p-value	0.2916[1]	0.1410[1]
		Difference V5-V2 (Tx-Px)	0.07±1.18	
		LS mean difference	0.07	
		p-value	0.7886[3]	
	Trunk	V2	40.39±6.76	42.19±6.06
		V5	39.94±6.87	42.20±6.16
		V5-V2	-0.45±1.81	0.01±1.40
		p-value	0.1167[1]	0.9647[1]
		Difference V5-V2 (Tx-Px)	-0.46±1.62	
		LS mean difference	-0.49	

		p-value	0.1765[3]	
	Android	V2	42.67±7.63	44.54±6.58
		V5	42.42±7.61	44.77±6.74
		V5-V2	-0.26±2.19	0.23±1.75
		p-value	0.4517[1]	0.4121[1]
		Difference V5-V2 (Tx-Px)	-0.48±1.99	
		LS mean difference	-0.52	
		p-value	0.2279[3]	
	Gynoid	V2	35.81±8.63	39.07±8.38
		V5	35.72±8.86	38.90±8.34
		V5-V2	-0.09±1.35	-0.16±1.50
		p-value	0.6752[1]	0.4891[1]
		Difference V5-V2 (Tx-Px)	0.08±1.43	
		LS mean difference	0.07	
		p-value	0.8401[3]	
Fat-free mass (g), mean±SD	Arm	V2	5259.83±1434.55	4825.59±1447.03
		V5	5221.40±1398.37	4864.32±1466.10
		V5-V2	-38.43±321.71	38.73±265.01
		p-value	0.7728[2]	0.4868[2]
		Difference V5-V2 (Tx-Px)	-77.16±295.07	
		p-value	0.4415[4]	
	Legs	V2	15562.10±3223.26	14749.71±3172.57
		V5	15316.62±3075.54	14747.10±3177.69
		V5-V2	-245.48±629.62	-2.61±508.51
		p-value	0.0155[1]	0.9739[1]
		Difference V5-V2 (Tx-Px)	-242.87±573.02	
		LS mean difference	-204.55	
		p-value	0.1023[3]	

	Trunk	V2	22465.10±3967.22	21025.29±3809.72
		V5	22276.95±4001.92	21006.56±3684.69
		V5-V2	-188.14±1230.11	-18.73±738.79
		p-value	0.6141[2]	0.8718[1]
		Difference V5-V2 (Tx-Px)	-169.41±1017.57	
		p-value	0.7987[4]	
	Android	V2	3233.17±688.73	3014.71±580.10
		V5	3151.40±654.83	2999.66±565.22
		V5-V2	-81.76±205.95	-15.05±137.82
		p-value	0.0138[1]	0.5925[2]
		Difference V5-V2 (Tx-Px)	-66.71±175.64	
		p-value	0.1329[4]	
	Gynoid	V2	7305.05±1578.33	6848.68±1493.16
		V5	7196.81±1545.12	6833.27±1474.53
		V5-V2	-108.24±268.91	-15.41±168.28
		p-value	0.0126[1]	0.5608[1]
		Difference V5-V2 (Tx-Px)	-92.82±224.92	
		LS mean difference	-71.70	
		p-value	0.1419[3]	

[1] p-value for the paired t-test

[2] p-value for the Wilcoxon signed rank test

[3] p-value for ANCOVA adjusted for baseline and sex

[4] p-value for the Wilcoxon rank sum test

Table S5. CT results at 12 weeks from baseline (PPS)

		BB-3 group (N=42)	Placebo group (N=41)
Total fat area (cm²), mean±SD	V2	359.07±60.96	378.02±59.20
	V5	359.73±66.35	389.58±74.95
	V5-V2	0.66±52.06	11.56±51.91
	p-value	0.9347[1]	0.1616[1]
	Difference V5-V2 (Tx-Px)	-10.90±51.98	
	LS mean difference	-12.78	
	p-value	0.2591[3]	
Subcutaneous fat area (cm²), mean±SD	V2	218.72±52.88	242.90±52.65
	V5	220.98±64.55	254.93±65.79
	V5-V2	2.26±44.79	12.03±41.32
	p-value	0.7456[1]	0.0697[1]
	Difference V5-V2 (Tx-Px)	-9.77±43.11	
	LS mean difference	-11.76	
	p-value	0.2246[3]	
Visceral fat area (cm²), mean±SD	V2	140.35±47.36	132.68±44.92
	V5	138.75±48.78	134.65±43.95
	V5-V2	-1.60±26.72	1.97±24.15
	p-value	0.7008[1]	0.6039[1]
	Difference V5-V2 (Tx-Px)	-3.57±25.48	
	LS mean difference	-2.29	
	p-value	0.6776[3]	
Visceral fat:subcutaneous fat ratio (VSR), mean±SD	V2	0.70±0.34	0.58±0.26
	V5	0.71±0.39	0.57±0.26
	V5-V2	0.01±0.19	-0.02±0.15
	p-value	0.5564[2]	0.0891[2]
	Difference V5-V2 (Tx-Px)	0.02±0.17	
	p-value	0.8163[4]	

[1] p-value for the paired t-test

[2] p-value for the Wilcoxon signed rank test

[3] p-value for ANCOVA adjusted for baseline and sex

[4] p-value for the Wilcoxon rank sum test

Table S6. Results of blood lipid levels at 12 weeks from baseline (PPS)

		BB-3 group (N=42)	Placebo group (N=41)
Total cholesterol (mg/dl), mean±SD	V1	198.05±32.62	201.41±36.59
	V5	188.81±31.41	196.05±35.21
	V5-V1	-9.24±23.83	-5.37±25.93
	p-value	0.0160[1]	0.1927[1]
	Difference V5-V1 (Tx-Px)	-3.87±24.89	
	LS mean difference	-4.41	
	p-value	0.3847[3]	
LDL cholesterol (mg/dl), mean±SD	V1	119.07±26.33	121.51±27.23
	V5	111.50±22.07	117.49±25.21
	V5-V1	-7.57±16.07	-4.02±16.89
	p-value	0.0040[1]	0.1349[1]
	Difference V5-V1 (Tx-Px)	-3.55±16.48	
	LS mean difference	-4.27	
	p-value	0.1889[3]	
Triglyceride (mg/dl), mean±SD	V1	150.83±83.53	145.15±80.41
	V5	149.38±73.95	135.41±64.38
	V5-V1	-1.45±84.08	-9.73±56.75
	p-value	0.9114[1]	0.6610[2]
	Difference V5-V1 (Tx-Px)	8.28±71.89	
	p-value	0.6325[4]	
HDL cholesterol (mg/dl), mean±SD	V1	53.50±10.82	54.98±10.44
	V5	50.10±8.90	51.98±9.79
	V5-V1	-3.40±6.16	-3.00±6.86
	p-value	0.0009[1]	0.0079[1]
	Difference V5-V1 (Tx-Px)	-0.40±6.52	
	LS mean difference	-0.60	
	p-value	0.6300[4]	
Leptin (mg/dl), mean±SD	V1	21.03±14.23	28.22±15.77
	V5	17.33±11.89	28.16±19.54
	V5-V1	-3.69±6.59	-0.06±11.92
	p-value	0.0004[2]	0.5793[2]
	Difference V5-V1 (Tx-Px)	-3.63±9.60	
	p-value	0.1425[4]	
Adiponectin (mg/dl), mean±SD	V1	6933.11±3818.28	8448.21±4762.79
	V5	6494.44±3091.42	8764.41±5787.27
	V5-V1	-438.67±2193.65	316.21±2706.40
	p-value	0.2496[2]	0.5113[2]

Difference V5-V1 (Tx-Px)	-754.87±2460.25
p-value	0.7882[4]

[1] p-value for the paired t-test
[2] p-value for the Wilcoxon signed rank test
[3] p-value for ANCOVA adjusted for baseline and sex
[4] p-value for the Wilcoxon rank sum test

Table S7. Adverse effects (SAS)

		BB-3 group (N=51)	Placebo group (N=49)
		n (%) [F]	n (%) [F]
AE		15 (29.41) [21]	14 (28.57) [19]
	(95% CI†)	(16.91, 41.92)	(15.92, 41.22)
	p-value[1]	0.9262	
ADR		0 (0.00) [0]	0 (0.00) [0]
	(95% CI†)	(0.00, 0.00)	(0.00, 0.00)
	p-value[1]	-	
SAE		0 (0.00) [0]	0 (0.00) [0]
	(95% CI†)	(0.00, 0.00)	(0.00, 0.00)
	p-value[1]	-	

Note: [F]=frequency

[1] Compared between groups; p-value for the Chi-square test (C) or Fisher's exact test (F)

Table S8. Changes in physical activity and dietary habits (PPS)

		BB-3 group (N=42)	Placebo group (N=41)
IPAQ Total Physical activity (MET-min/week)	V2	2401.50±1955.78	2848.59±3562.14
	V5	4151.02±5655.05	2808.20±3294.04
	V5-V2	1749.52±4582.37	-40.39±2645.99
	p-value	0.3412[2]	
IPAQ Total Energy expenditure (Kcal/week)	V2	3065.91±2694.03	3653.76±4955.91
	V5	5257.72±7531.41	3509.73±4546.55
	V5-V2	2191.81±5915.85	-144.02±3317.14
	p-value	0.3458[2]	
Dietary survey (Kcal)	V2	1883.56±573.85	1827.53±657.96
	V5	1811.72±440.19	1721.49±545.25
	V5-V2	-71.84±579.75	-106.03±704.08
	p-value	0.8734[2]	

[1] p-value for the two-sample t-test, [2] p-value for the Wilcoxon rank sum test

The International Physical Activity Questionnaires (IPAQ) was used to assess physical activity, while the 24-hour dietary recall method was used to assess dietary habits.

Table S9. Blood test results (SAS)

		BB-3 (N=51)	Placebo (N=49)
WBC ($10^3/\mu l$), mean±SD	V1	6.12±1.35	6.21±2.11
	V5	5.93±1.09	6.10±1.71
	V5-V1	-0.09±0.85	-0.16±1.45
	p-value	0.4665[1]	0.9662[2]
	p-value	0.5423[4]	
RBC ($10^3/\mu l$), mean±SD	V1	4.64±0.44	4.66±0.42
	V5	4.91±0.45	4.80±0.44
	V5-V1	0.22±0.28	0.13±0.31
	p-value	0.0000[2]	0.0008[2]
	p-value	0.3370[4]	
Hemoglobin (g/dl), mean±SD	V1	13.94±1.53	14.06±1.45
	V5	14.67±1.43	14.49±1.24
	V5-V1	0.56±0.78	0.36±0.75
	p-value	0.0000[2]	0.0006[2]
	p-value	0.7163[4]	
Hematocrit (%), mean±SD	V1	41.25±4.01	41.62±3.93
	V5	43.25±3.85	42.85±3.44
	V5-V1	1.57±2.27	1.00±2.63
	p-value	0.0000[2]	0.0024[2]
	p-value	0.6642[4]	
Platelets ($10^3/\mu l$), mean±SD	V1	251.80±58.64	268.04±65.58
	V5	268.07±65.62	273.79±58.18
	V5-V1	18.80±36.03	8.53±40.91
	p-value	0.0000[2]	0.0165[2]
	p-value	0.5477[4]	
Glucose (mg/dl), mean±SD	V1	93.22±9.52	96.12±12.90
	V5	92.47±9.31	90.79±14.29
	V5-V1	-1.53±10.48	-5.95±11.03
	p-value	0.3316[1]	0.0010[1]
	p-value	0.0572[3]	
BUN (mg/dl), mean±SD	V1	14.09±3.28	14.11±3.75
	V5	13.59±3.50	13.17±3.54
	V5-V1	-0.48±3.36	-0.90±3.48
	p-value	0.3479[1]	0.0963[1]
	p-value	0.5599[3]	
Creatinine (mg/dl),	V1	0.82±0.13	0.85±0.15
	V5	0.81±0.15	0.81±0.18

mean±SD	V5-V1	-0.02±0.10	-0.03±0.11
	p-value	0.1702[1]	0.0363[1]
	p-value	0.5349[1]	
Total protein (g/dl), mean±SD	V1	7.40±0.35	7.42±0.32
	V5	7.27±0.38	7.21±0.33
	V5-V1	-0.14±0.39	-0.21±0.37
	p-value	0.0057[2]	0.0006[1]
	p-value	0.4515[4]	
Albumin (g/dl), mean±SD	V1	4.26±10.27	4.29±0.24
	V5	4.20±0.06	4.28±0.25
	V5-V1	-0.07±0.31	-0.03±0.23
	p-value	0.0990[2]	0.3357[1]
	p-value	0.5856[4]	
Total bilirubin (mg/dl), mean±SD	V1	0.81±0.37	0.79±0.31
	V5	0.88±0.44	0.73±0.28
	V5-V1	0.03±0.26	-0.08±0.32
	p-value	0.4243[1]	0.1043[1]
	p-value	0.0740[3]	
AST (UL), mean±SD	V1	23.69±6.30	25.35±10.34
	V5	24.33±6.62	29.02±36.29
	V5-V1	0.84±6.18	4.74±36.42
	p-value	0.3640[1]	0.6786[2]
	p-value	0.6725[4]	
ALT (U/L), mean±SD	V1	21.73±9.69	25.80±18.74
	V5	25.76±13.83	25.74±16.31
	V5-V1	3.89±10.35	1.26±13.27
	p-value	0.0239[2]	0.4360[2]
	p-value	0.3215[4]	
γ-GT (U/L), mean±SD	V1	31.00±27.77	28.47±22.58
	V5	31.64±30.64	27.42±19.90
	V5-V1	-0.78±12.26	-0.23±5.96
	p-value	0.3109[2]	0.6757[2]
	p-value	0.6363[4]	

[1] p-value for the paired t-test
[2] p-value for the Wilcoxon signed rank test
[3] p-value for the two-sample t-test
[4] p-value for the Wilcoxon rank sum test

Table S10. Urine test results (SAS)

		Week 12/baseline (Week 1)	Normal, n (%)	Abnormal, n (%)
**Specific gravity**	BB-3 group (N=51)	Normal, n (%)	34 (66.67)	5(9.80)
		Abnormal, n (%)	4 (7.84)	2(3.92)
		p-value	0.7389[1]	
	Placebo group (N=49)	Normal, n (%)	35 (71.43)	4(8.16)
		Abnormal, n (%)	2 (3.92)	2(3.92)
		p-value	0.4142[1]	
**pH**	BB-3 group (N=51)	Normal, n (%)	45 (88.24)	0 (0.00)
		Abnormal, n (%)	0 (0.00)	0 (0.00)
		p-value	-	
	Placebo group (N=49)	Normal, n (%)	43 (87.76)	0 (0.00)
		Abnormal, n (%)	0(0.00)	0 (0.00)
		p-value	-	
**Protein**	BB-3 group (N=51)	Normal, n (%)	30 (58.82)	7 (13.73)
		Abnormal, n (%)	7 (13.73)	1 (1.96)
		p-value	1.0000[1]	
	Placebo group (N=49)	Normal, n (%)	32 (65.31)	7 (14.29)
		Abnormal, n (%)	2 (3.92)	2 (3.92)
		p-value	0.0956[1]	
**Glucose**	BB-3 group (N=51)	Normal, n (%)	45 (88.24)	0 (0.00)
		Abnormal, n (%)	0 (0.00)	0 (0.00)
		p-value	-	
	Placebo group (N=49)	Normal, n (%)	42 (85.71)	1 (2.04)
		Abnormal, n (%)	0 (0.00)	0 (0.00)
		p-value	-	

**Blood (RBC)**	BB-3 group (N=51)	Normal, n (%)	27 (52.94)	8 (15.69)
		Abnormal, n (%)	3 (5.88)	7 (13.73)
		p-value	0.1317[1]	
	Placebo group (N=49)	Normal, n (%)	24 (48.98)	7 (14.29)
		Abnormal, n (%)	3 (5.88)	9 (17.65)
		p-value	0.2059[1]	

[1] *p*-value for McNemar's test

Table S11. Abbreviations and glossary of terms

ADR	Adverse drug reaction
AE	Adverse event
ALP	Alkaline phosphatase
ALT	Alanine aminotransferase
ANCOVA	Analysis of covariance
AST	Aspartate aminotransferase
BB-3	*Bifidobacterium breve* B-3
BUN	Blood urea nitrogen
CFU	Colony-forming units
CIM	carbohydrate-insulin model
COVID-19	Coronavirus disease 2019
CRP	C-reactive protein
CT	Computed tomography
DALY	the global deaths and disability-adjusted life years
DEXA	Dual energy x-ray absorptiometry
EBM	energy balance model
ESR	Erythrocyte sedimentation rate
FAS	Full analysis set
GBD	Global Burden of Disease Study
γ-GT	Gamma-glutamyltransferase
HCG	Human chorionic gonadotropin
HDL-C	High density lipoprotein-cholesterol
IPAQ	International Physical Activity Questionnaires
IRB	Institutional review board
IWRS	Interactive web response system
LDL-C	Low-density lipoprotein-cholesterol
MET	Metabolic equivalent of task
PPS	Per protocol set
RBC	Red blood cell
SAE	Serious adverse event
SAS	Safety analysis set
TG	Triglyceride
VSR	Visceral fat area/subcutaneous fat area ratio
UNL	Upper normal limit
WBC	White blood cell
